# Personal and relational experiences on meditation journeys following developmental trauma: An IPA study of adults who experienced an inconsistent evolved developmental niche

**DOI:** 10.1111/papt.70018

**Published:** 2025-10-21

**Authors:** Anna‐Maria Frastali, Adhip Rawal

**Affiliations:** ^1^ School of Psychology, University of Sussex Sussex UK

**Keywords:** childhood trauma, defence mechanisms, emotional difficulties, evolved developmental niche, meditation, relational difficulties

## Abstract

**Objectives:**

In recent decades, research has increasingly highlighted the devastating effects of childhood trauma and relational processes that violate human development. However, the unique dynamics of such early‐life deprivations in adults who practice meditation, a context where the complexity of such wounding (and healing) may become apparent, remains underexplored. The objectives of this study were to explore how individuals with a history of inconsistent evolved developmental niche (EDN) care experience their meditation journeys, and to understand the emotional and relational processes that emerge in this context.

**Design:**

A qualitative design using interpretative phenomenological analysis (IPA) was adopted to capture participants' lived experiences.

**Methods:**

Six adults with a history of inconsistent EDN care and at least 3 months of regular meditation practice participated in semi‐structured interviews. Transcripts were analysed using IPA to identify key experiential themes.

**Results:**

Three themes emerged from the data: (1) ‘An inconsistent EDN in childhood left lasting emotional and relational wounds into adulthood’, (2) ‘Meditation eased inner turmoil but revealed different ways of dealing with emotions and the body’, and (3) ‘Meditation offered a space to explore relating to the self and others, areas complicated by their inconsistent EDN’. Findings illustrated the inheritance and development of immature defence mechanisms alongside emotional and relational challenges. Participants described feelings of increased calmness and closeness with others as benefits of meditation, though for some, pre‐existing difficulties persisted or were amplified.

**Conclusion:**

These results raise awareness of the long‐term effects surrounding unresolved traumatic dynamics and the importance of delivering trauma‐informed care. They also highlight the potential of meditation to uncover psychological dynamics and experiences rooted in early life which have the emotional potency to pose risks to individuals coming into contact with them.

## INTRODUCTION

Over the past decade childhood trauma has received increased interest in research and popular culture due to its profound and lasting impact on individuals' mental, emotional, and physical well‐being. Its definition is unclear; some researchers define it based on its long‐term detrimental effects (Terr, 2003), some based on events experienced (Bernstein et al., 2003), and some based on a combination of both (Van der Kolk, 2014). Technically, trauma occurs when experiences lead to intense negative emotions and physical symptoms that persist beyond initial exposure (Bartlett & Sacks, 2019). In practice, ‘childhood trauma’ is used loosely and interchangeably with ‘adverse childhood experiences’ (ACE) in research (e.g. Oral et al., 2016; Whitfield et al., 2005), the latter being widely used in psychology to assess childhood exposure to experiences such as abuse and neglect which *can* be traumatic (Gilgoff et al., 2020). Regardless of whether they are defined as ‘traumatic’ or not, ACEs can have detrimental effects, being associated with depressed affect, anxiety, sleep disruption, substance abuse as well as suicide attempts in adulthood (Merrick et al., 2017; Poole et al., 2017).

Recent findings from anthropology, neuroscience, and developmental psychology have added nuance to our understanding of childhood trauma, giving rise to the concept of the evolved developmental niche (EDN) (Narvaez et al., 2012). The EDN proposes that humans evolved as social mammals over 30 million years ago with parenting practices that optimise species development, including soothing perinatal experiences, long‐term breastfeeding, a supportive communal network with responsive caregivers, social play and connection to nature (Tarsha & Narvaez, 2019). This perspective presents human children as critically dependent on prolonged, sensitive caregiving and differs from that of ACE in two ways. First, it considers both positive and negative childhood experiences, arguing that positive experiences can act in a protective manner while their absence can be damaging (Tarsha & Narvaez, 2019, 2022). Secondly, the EDN focuses on the relational, embodied and emotional aspects of a person's existence including affection in child–carer relationships, the home environment and the child's sense of belonging, seeing individuals as embedded within and extended by relationships from the very beginning (Narvaez et al., 2016). In contrast, the ACE's approach to measure negative events that happened *to* someone, does not capture these ontological dimensions with the same nuance and pays less attention to how a person felt and responded to experiences in their childhood. The difference is essential as a recent study found that a consistent EDN can act as a buffer against experiencing ACEs by supporting vagal adaptability, which is essential in emotion regulation and resilience to stress (Porges, 1992; Tarsha & Narvaez, 2022).

The relational focus of the EDN presents an inherent link to attachment. Secure attachment, a healthy bond formed between child and primary caregiver, is marked by trust in the caregiver's responsiveness and availability, forming a foundation for emotional regulation and a positive sense of self (Ainsworth et al., 1978; Bowlby, 1982; Mikulincer, 1995). By contrast, insecure attachment can lead to dysregulated stress responses, reduced self‐esteem and difficulties in forming and maintaining healthy relationships throughout adulthood (Maunder & Hunter, 2008; Sroufe et al., 2009). A consistent EDN has been associated with secure attachment, social engagement, empathy and self‐regulation (Narvaez et al., 2013, 2016; Tarsha & Narvaez, 2022). An inconsistent EDN, on the other hand, is associated with insecure attachment, anxiety and depression, moral incapacity, and high social opposition and withdrawal (Narvaez et al., 2016).

The association between the EDN and self‐regulation is unsurprising as emotion regulation develops through early carer–child interactions (Fonagy et al., 2018). An emotionally responsive parent can share and co‐regulate the child's anxieties and thus provide the structure to build emotion regulation, while the absence of such parenting can contribute to developing maladaptive defence mechanisms to manage difficult emotions (Lemma, 2015). Defence mechanisms are automatic reactions to internal or external stressors. Neurotic and immature defences, such as repression, idealisation, and dissociation, incrementally hinder healthy interpersonal relationships and distort reality (Békés et al., 2021; Di Giuseppe & Perry, 2021). Recent studies show a correlation between immature defences and high exposure to ACEs/childhood trauma, insecure attachment, high anxiety and depression symptoms (Ferrajão et al., 2024; Prunas et al., 2019; Uygur et al., 2022). Even though there are no studies to date on EDN and defence mechanisms, since an inconsistent EDN implies emotionally unresponsive parenting and potential exposure to ACEs, it is highly possible that people with an inconsistent EDN use immature defence mechanisms and emotion regulation strategies to deal with the (unexpected) reality of species atypical care.

Based on these findings, exposure to an inconsistent EDN would likely pose a threat to self‐development and lead to emotional and/or relational challenges, and therefore a need to alleviate distress. A popular avenue, nowadays, for those seeking wellness is that of meditation – and indeed, alleviating mental health issues or stress has been reported as the main motivation to start meditation (Jiwani et al., 2022). Meditation has become increasingly practiced in the Western world, with over 5% of adults in the US practising meditation (Cramer et al., 2016) and 15% of adults in the UK practising mindfulness meditation today (Simonsson et al., 2021). Meditation generally involves the cultivation of awareness, in formal or informal settings, and different styles have been identified (Sparby & Sacchet, 2022). Meditation styles can be divided into three categories, based on cognitive mechanisms at work (Dahl et al., 2015). The ‘attentional’ focuses on observing experience, the ‘constructive’ cultivates virtues such as kindness and compassion, and the ‘deconstructive’ encourages the practitioner to investigate the nature of their conscious experience. Practices tend to contain elements of all three styles but use one as their primary style.

Studies have reported multiple benefits associated with practising meditation. Mindfulness, an attentional practice, can lead to reduction of stress, anxiety, depression, distress and improvement of quality of life (Khoury et al., 2015). Loving‐kindness, a constructive practice, has been found to increase compassion, resilience, social connectedness and to decrease negative interpersonal attitudes and self‐focus (Hutcherson et al., 2008; Seppala et al., 2014; Zhou et al., 2023). The deconstructing practice of vipassana has been associated with personality changes in responsibility, authenticity, self‐compassion and self‐acceptance (Crescentini & Capurso, 2015). These findings may explain why meditation appeals to individuals with trauma histories seeking healing, as meditation encourages self‐reflection and self‐acceptance, mirroring trauma therapy practices (Van der Kolk, 2014).

While meditation is widely available to everyone today, little is known about the individual paths and experiences of people practising in the community. Meditation benefits are mainly reported following clinical interventions, yet, there is significant lack of studies in community where various forms of meditation are available. Interventions may offer precise, quantifiable results, but it is not clear whether these findings apply to those practicing in real life settings, or what the individual lived experience and journey looks like. This is of particular importance as recent studies have revealed that meditation can also have adverse effects. Meditation‐related adverse effects (MRAE) can be increased anxiety, traumatic‐re‐experiencing, exacerbation of pre‐existing mental health issues such as depression and emotional sensitivity and a loss of sense of self and embodiment (Goldberg et al., 2022; Lomas et al., 2015). Such experiences are far from rare: 80% of a non‐clinical population of novice and expert meditators have reported MRAEs, often leading to severe levels of distress and impairment (Britton et al., 2021; Lindahl et al., 2017). Particularly relevant to this study are findings that individuals with childhood trauma or adversity are more susceptible to MRAEs (Goldberg et al., 2022; Lindahl et al., 2017).

Our aim was to gain an insight into emotional and relational experiences of individuals with a meditative practice and history of EDN‐inconsistent care. No study, to our knowledge, has connected childhood and meditation experiences in this way. Exploring such experiences is important as the practice of meditation may bring to the surface developmental wounds that might otherwise go unnoticed. Although the prevalence of inconsistent EDN is undocumented, half a million children in the UK suffer abuse or neglect by their caregivers annually (Harker et al., 2013). Moreover, the evolution of our societies appears to counter the necessities of the ‘human nest’: The individualistic cultural orientation conflicts with relational sensitivities and offers less and less a communal sense of stability and belonging. Due to this, trauma is potentially passed on over generations and deeply present in the way individuals are raised. It is crucial, therefore, to provide nuanced, trauma‐informed care, and this study aims to contribute to the knowledge in this field. We employed Interpretative Phenomenological analysis (IPA) to explore one research question: *Are the emotional and relational effects of an inconsistent EDN reflected in a meditation journey?*

## METHODS

### Design

IPA seeks to understand participants' lived experiences, employing a double hermeneutic: making sense of the participants' experience as they try to make sense of their experience (Smith, 2007). IPA was chosen due to the experiential focus of this study, as qualitative data capture complex nuances, providing a more holistic comprehension of personal experiences. Additionally, given the lack of prior research on this topic, IPA's focus on the immediate experience of participants and the development of detailed experiential narratives is helpful in grounding interpretative claims of researchers. As such, IPA reveals and maintains proximity to the complexities of lived experience which guide the direction of analytic narratives. While there is no guarantee that these unfold without researcher biases, we stayed close to these principles of IPA and framed the research as openly as possible. We also allowed time and space for multiple readings and interrogations of transcripts so that new understandings and limitations of our perspectives could emerge. In writing the results, the first author proposed an initial thematic structure. The second author then analysed the transcripts independently, with knowledge of the initial thematic structure, and suggested refinements to the number and presentation of themes. Once these were written‐up, the researchers came together again to question the thematic structure and interpretations.

Empathic listening and rapport‐building were prioritised; thus, only one researcher was present in interviews to ensure comfort and trust. Interviews, lasting 1–2 hours, were conducted one‐on‐one in private, quiet settings by either the first author or another postgraduate student from the University of Sussex. A semi‐structured interview guide was developed through reflective discussions between the authors, covering key areas while allowing flexibility for in‐depth responses. To ensure quality and appropriateness of the interview guide (Table 1), a pilot interview was conducted as well as a debriefing session with the second author. The interview started with Question 1 as a warm‐up, with other questions asked responsively, following each participant's natural flow of thought. Prompts were prepared in case participants needed further guidance. In the end, participants had the opportunity to address anything they wanted and was not already covered. Interviews were audio recorded and then transcribed verbatim.

**TABLE 1:** Interview guide.

1. What meditation practices have you followed and for how long? Have you ever attended a meditation retreat?
2. Can you tell me about your emotional experiences during meditation practice?
3. Can you tell me about any changes you've noticed in the way you perceive yourself?
4. How has your practice impacted your relationships with other people?
5. How do you think your childhood experiences impacted your experiences throughout your meditation practice in any way?
6. How would you describe your meditation experience overall?

### Participants

In qualitative research in general, and specifically in IPA, homogeneity of experience, meaning that participants have experienced the studied phenomenon, is desired (Larkin et al., 2021). Here, homogeneity was based on experiences of meditation and EDN‐inconsistent care. An online survey gathered background information, including meditation practice and childhood history. The survey also included a free text box for participants to describe their childhood in their own words and it had two screening questions to ensure that participants were adults and did not have a diagnosed mental health condition or disability severely impacting daily life.

Recruitment was conducted in two stages. First, potential participants filled in the online survey. Sampling was voluntary as due to the intimate nature of conducting IPA interviews it was important to ensure that people participate freely and wilfully. The study was advertised through posters at places that meditators frequent, such as local meditation centres and yoga studios, as well as the University of Sussex campus. About 13 people expressed interest in participating and 11 people filled in the online survey. Second, participants meeting these criteria were invited for an interview: (1) At least 3 months of meditation experience and practising several times per week guided by the fact that meditation interventions usually tend to last 8 weeks (e.g. Juul et al., 2020; Kohls et al., 2019); (2) We reviewed responses to the childhood questionnaire and personal entries in the free text box, paying attention to the content and style of testimonies that spoke to the dimensions of the EDN including the loss of empathy, emotional deprivation, and/or relational neglect to help us explore our research question. Six participants were selected. Small numbers of participants are common and desirable in IPA studies; a small sample size allows for a deeper examination of similarities and differences between participants, which is the aim of IPA rather than generalising to the wider population (Pietkiewicz & Smith, 2014).

Participants were aged between 22 and 57 (*M* = 35.00), with equal gender distribution, some ethnic diversity, and lower to middle socio‐economic backgrounds. Participants were not compensated but travel expenses were covered. Pseudonyms were used throughout the manuscript to protect identities. Table 2 presents participant demographics and meditation practice details.

**TABLE 2:** Demographic details of participants.

Participant pseudonym	Gender	Age	Ethnicity	Income	Length of meditation practice	Meditating frequency	EDN score	Meditation practice/practiced in a religious or secular context
Viv	Female	22	Indian	£10,000–£24,999	1–2 years	Daily, 60 mins	19.3	Bhakti Yoga/Hinduism
Dolores	Female	27	White Eastern European	£25,000–£49,999	+1 year	+ 3 days per week, 10–30 min	24.2	Various types instructed in yoga classes including pranayama/secular
Theo	Male	32	White British	£25,000–£49,999	+2 years	Daily, 10–30 mins	20.8	Mindfulness, Eckhart Tolls, Zen Koan/secular
Tony	Male	33	African	£25,000–£49,999	+2 years	Daily, 10–30 mins	24.4	Mindfulness/secular
Cleo	Female	39	White British	£25,000–£49,999	+2 years	Daily, ≤10 min	18.1	Mindfulness, vipassana/secular
Bill	Male	57	White British	£25,000–£49,999	+2 years	Daily, 10–30 mins	25.9	Mindfulness and loving‐kindness/Buddhism

### Measures

To assess meditation practice, the online survey included some general questions regarding historic length, current frequency, and duration of practice. The type of meditation practiced was not pre‐assessed and was asked during the interview.

Childhood experiences were assessed using the EDN History questionnaire (Narvaez et al., 2016). The questionnaire refers to experiences before the age of 18, covering family climate, parental affection, playing and breastfeeding, with questions such as ‘How much support and affection did you receive in your childhood?’ Answers are presented in a Likert‐scale from ‘Very little or not at all’ to ‘Very much’. Questions on breastfeeding and circumcision are dichotomous (yes/no). Scoring followed the standard Narvaez syntax. Scores range from 8–36 with scores below 25 reflecting a more inconsistent EDN that may include emotional or physical abuse, lack of affectionate touch and play, a non‐responsive social environment, and a negative home climate. Selected participants scored between 18 and 26. Participants could also describe challenging aspects of their childhood in their own words in an open‐text box, providing a crucial context for IPA, as the questionnaire may feel restrictive or insufficient.

### Data analysis

Interviews were listened to at least once for familiarisation, while transcripts were read multiple times to ensure immersion in each narrative. Analysis consisted of the following steps: capturing initial thoughts separately, analysing the text closely through exploratory comments and highlighting the transcript, identifying initial themes per participant, matching each participant's quotes to those themes and finally finding overarching themes that fit most narratives (Larkin et al., 2021). To ensure quality and validity, the analysis followed four key principles recommended for IPA research: creating a rich and engaging narrative, exploring the deeper personal meanings of participants' experiences, carefully examining the data in detail, and recognising both shared patterns and individual differences across participants (Nizza et al., 2021). After a brief opening description, each sub‐theme is therefore developed by purposefully chosen quotations that illustrate the material that emerged in the interviews, alongside an analytic commentary that connects these quotes into a narrative focused on participants' meaning‐making, aided by our reflections on their lived experience. We intended to uncover experiential and existential dimensions that speak to the broader and deeper significance of participants' experiences. Paying attention to convergences and the uniqueness of experiences was important to the validity and enrichment of themes.

### Ethical issues

Participants were informed of the study's purpose and gave written consent via the online survey. Data were securely stored online. During interviews, participants were reminded of the purpose of the study and their right to withdraw at any time or refuse response. Given the potential for sensitive discussion, participants were debriefed and encouraged to raise any concerns afterward. Ethical approval for this study was granted by the University of Sussex.

## RESULTS

The analysis identified three overarching themes: ‘An inconsistent EDN in childhood left lasting emotional and relational wounds into adulthood’; ‘Meditation eased inner turmoil but revealed different ways of dealing with emotions and the body’, with sub‐themes ‘Some used meditation to face and release painful feelings; others to suppress or avoid them’, and ‘For some, emotions were processed through the body; others kept the body distant, seeing it as temporary or secondary’; and ‘Meditation offered a space to explore relating to the self and others, areas complicated by their inconsistent EDN’, with sub‐themes ‘Many struggled with self‐love and self‐compassion’, and ‘While participants found that meditation increased empathy, difficulties with appropriate relational closeness were presented’. The first theme reflects the impact of participants' inconsistent EDN. The second explores how meditation and childhood experiences inform how they calm inner turmoil. The third outlines how these factors influence participants' relationships with themselves and others (Table 3).

**TABLE 3:** A Description of the themes and sub‐themes identified with examples from the interviews.

Theme	Sub‐theme	Description	Example
An inconsistent EDN in childhood left lasting emotional and relational wounds into adulthood	N/A	How participants experienced the impact of having grown up with inconsistent EDN care	‘*ever since I was nine, I kind of fended for myself*’ ‘*Because of what my relationship was like with my parents*…*I don't give anything to myself*’
Meditation eased inner turmoil but revealed different ways of dealing with emotions and the body	Some used meditation to face and release painful feelings; others to suppress or avoid them	How participants managed their inner turmoil in their meditation practice	‘*all feelings and sensations are welcome*’ vs. ‘*Go down the road at frustration and anger and*… *Get rid of all that*’
	For some, emotions were processed through the body; others kept the body distant, seeing it as temporary or secondary	How participants experienced and related to their bodies as reflected in their meditative practice	‘*the body has an intelligence that perhaps supersedes conscious intelligence*’ vs. ‘*my body's just like a vehicle*’
Meditation offered a space to explore relating to the self and others, areas complicated by their inconsistent EDN	Many struggled with self‐love and self‐compassion	How participants explored their relationship to themselves through the processes of self‐love and self‐compassion	‘*I think the self‐love thing is still burning in my mind […] I neglected my own happiness*’ ‘*had the kind of capacity to comfort that boy and tell him that it's OK, you feel safe*’
	While participants found that meditation increased empathy, difficulties with appropriate relational closeness were presented	How meditation brought participants closer to others but they still maintained their distance	‘*…if I was well connected to my practice I wouldn't be dependent on my friends and family, or my relationships*’

### An inconsistent EDN in childhood left lasting emotional and relational wounds into adulthood

Participants shared varied experiences inconsistent with the EDN, largely characterised by neglectful, unresponsive, or emotionally abusive family environments.

Viv's childhood was marked by separation and emotional distance from her parents, who left her in India at the age of 4 with extended family while they moved to the UK. She recalls a happy life in India, ‘I loved living with my grandparents and like the rest of the family, I was so happy’. However, her life changed when she was brought to the UK at the age of 8, a move she did not want: ‘I was more upset when I came to the UK, because I didn't want to leave them’ (her extended family in India). Even before this separation, her relationship with her parents felt detached: ‘When they (her parents) came back from work, I wouldn't wanna go back… my house was my grandma's house in my head’. Viv felt discouraged to express anger, explaining she never had ‘the right to be angry…Don't be angry or. You're like a bad kid’. Her mother, meanwhile, is described as emotionally volatile: ‘(Mum) was so stressed out even if I did the tiniest thing that wasn't right or didn't tick the box, then I'd get told off for it’. Viv recalls that ‘I just didn't feel comfortable enough to like, share my emotions with mum because I knew how stressed she was already and I was scared’. This emotional disallowance persists today: ‘…if I feel extreme sadness. I just forget it… My brain just… puts it in a place where I don't wanna face it’, and ‘Even if I tried to get angry, I don't know the emotion’. Viv today looks back by trying to understand and excuse her parents' behaviour: ‘Maybe I shouldn't be mad at my parents, you know, they had to come and earn…’, adding that ‘most dads don't (show emotions)’ and that her mum ‘had so much of her own trauma’.

Bill, similarly, experienced emotional neglect and parental absence: ‘My parents were hardly ever there (laughs)’. His father had a ‘violent temper’, creating a ‘toxic’ home environment: ‘nearly every day, my dad would be shouting at my mother making lots of allegations’. Bill felt unable to express his emotions: ‘… when you're an adolescent you're not able to talk about your feelings… sort of shut these down’, and ‘I didn't really feel I had the right to express anger’. Like Viv, he tries to sympathise with his father: ‘…there wasn't really a lot of access to any sort of psychology, understanding, or self‐development… but I think it's quite painful for him… He's from that generation where as a man you couldn't really admit to flaws’. To cope with the emotional toll of his childhood, Bill reframes his memories, explaining: ‘… you can choose how you want to look at things in, like your memories of childhood… with the positive lens you can actually just really appreciate how much love and care… Your memories aren't inherently real’.

Theo described an unavailable father, saying ‘My dad has always been quite a sort of peripheral figure, just like quite distant’ and an enmeshed relationship with his mother: ‘So she basically raised me, but that kind of closeness also, there's like being quite suffocating to me’. His parents' bitter divorce exposed him to emotional conflict: ‘constantly ranting about each other to me in really unforgiving, brutal terms’, showing a lack of awareness of ‘what they understood or didn't understand to be appropriate for a child's ears’. He noted that such environments compel children to suppress anger to survive: ‘your survival depends on your parents…it's only when you have some security of your own and encouragement and a good holding environment that you can start to explore some of those unmet buried feelings’. Today, he still feels triggered by his mother's behaviour: ‘using me as a kind of emotional crutch. Any number of things that can kind of bring you back to kind of childhood state where you were kind of asked to do too much emotional heavy lifting’. As an adult, Theo has distanced himself from his parents to protect his well‐being: ‘…Just to give me a bit more space to grow into’. Reflecting on his relationship with his father, he shares: ‘I then, tried to … make a kind of last‐ditch attempt to salvage something… I decided it wasn't my job to get him to understand, or if he ever could’.

Dolores grew up in Poland, where: ‘we don't really talk about emotions in Poland, like, that's a subject that is being avoided at all costs, especially with my parents’. Like Viv, she felt pressure to be: ‘the perfect child and always be smiling. You could never be angry or crying or whatever’, which led her to ‘detach myself from my emotions… so I couldn't be fully myself’. Moving to the UK helped her distance herself from her ‘emotionally abusive’ father: ‘I'm just going to buy one way ticket and I'm not going to come back’. However, her early abuse impacted how she related to others, ‘I was scared that someone else can treat me the same if I triggered them’. Today, she acknowledges a tendency to ‘intellectualise your feelings, so I don't like to feel them’. Reflecting on her childhood she recognises the value of ‘good stuff in my life… like with my grandparents. That's why I am who I am’.

Tony described a childhood marked by physical abuse and family ostracism due to his decision not to attend church: ‘I was beaten up a lot, not because I hurt anyone. But because I said no to their religion’. He recalls that ‘dad was not around’ while his relatives told him, ‘I know you gonna end up in jail because you don't get to church’. As a result, he felt a withdrawal of love: ‘They wouldn't show you love… we'll show you care. But on a minimal level and then we will show the others care because they listen to us’. This family rejection eventually led to experiences of ‘depersonalisation’: ‘you feel you are so distant from your body. Like when you experience yourself from the outside you just feel the form of disconnection from you and your body’. He also described difficulties in feeling joy: ‘my own being happy takes a lot of effort to achieve’. Reflecting on the broader impact of trauma, he noted: ‘I feel what the trauma does to people… especially to young black male is that they become aggressive later on… They transfer this hatred to other people… so they might likely do something stupid that they might probably end up in jail…’ Today, he tries to understand his family's behaviour: ‘You could see how childish they were… they are not necessary (sic) evil’.

Cleo described a chaotic childhood caused by her mother's instability: ‘When I was a child, chaos and uncertainty was a huge trigger for me’, and ‘a big part of her personality is we don't know which person she is. She drank too much’. Cleo recalled a traumatic event that remains ‘a sticking point of life for me’, ‘I was really battered afterwards and it was horrendous’. Her survival depended on self‐reliance, explaining: ‘ever since I was nine, I kind of fended for myself’. Although she maintains a relationship with her mother, Cleo works hard to set boundaries: ‘I've done so much acceptance and trying to find the good in that… I would never dream of taking my mum to France’, ‘I don't *need* (emphasis) to do that. I'm choosing to do it so now with all my work… I choose not to react to situations with her. I choose not to go back to being a teenager and try to stay above it’, yet a part of her seemed to want to avoid this trip: ‘Hopefully she can't come’. This ambivalence manifested in other relationships too: ‘I'm an island. And sometimes I need to dock onto the mainland for a little bit, and that's because what would happen, if an issue arises…’, perhaps carrying a sense of instrumentality towards others.

### Meditation eased inner turmoil but revealed different ways of dealing with emotions and the body

Participants commonly struggled with a ‘stream of concern and thoughts’ leading to anxiety, sadness, and depression. All except one, who was in psychotherapy, began meditating to manage these intense feelings. They described meditation as making them ‘more calm’ and that their anxiety ‘just goes’. This theme examines how participants found calm and relief through meditation.

#### Some used meditation to face and release painful feelings; others to suppress or avoid them

All participants processed their thoughts similarly, by noticing and letting them go. Tony described that before meditation he would ‘jump on the thought’ and ‘start feeding’ it while now he ‘allows’ it to ‘flow in and flow out’. Dolores similarly learnt not to ‘dwell’ on her thoughts, seeing them as ‘just a thought’.

However, participants differed in how they processed emotions. Theo, Tony and Dolores welcomed all emotions, enabling awareness and eventual release. Theo described meditation as ‘the window which is giving me access to my kind of inner landscape’ where ‘all feelings and sensations are welcome’. Similarly, Tony expressed that he is no longer ‘scared to feel any emotion’ and now he ‘can sit down with myself and feel the pain’. This acceptance of emotions often led to intense emotional release. Theo shared that he might be ‘crying for 20 minutes’, and that after you ‘opened yourself’ to difficult emotions you can ‘release them and that is an inherently joyful thing’. Tony describes it as a ‘digestion took place in my heart’, a metaphor suggesting an accumulation of unprocessed emotions that meditation allowed him to process in an embodied manner.

In contrast, Bill and Viv, who practice meditation with ties to Buddhism and Hinduism respectively, viewed meditation as a way to cleanse ‘negative’ emotions, approaching it as a spiritual practice to overcome such feelings. Bill described it as going ‘down the road at frustration and anger and… Get rid of all that’. While he expressed a goal of letting go of negative emotions, throughout the interview he struggled to articulate his emotions during meditation, responding in fragmented sentences:

obviously in life, if you go through a difficult time, a traumatic event, like splitting up with a girlfriend and then you know… that can. And the emotions from that will. You know, it's like, OK, yeah, that I thought I'd dealt with that, but still. I'm still… Experiencing a little bit of pain. So sometimes it (meditation) does make you more aware of that… underlying dissatisfaction or…?

This series of incomplete sentences reveals a difficulty, or resistance, in accessing his emotional world. His switch to present tense and first‐person narrative (‘I'm still’) suggests a past ‘traumatic event’ that remains unprocessed. Perhaps, fully confronting it would be too painful, so he diminishes it as ‘a little bit of pain’.

Viv, too, saw meditation as a practice of clearing “negative” emotions, guided by religious values tied to purity and morality. During an ideal meditation period, which she called “the best six months of my life,” and ‘transcendental’, she felt free of ‘pride, anger, greed, lust, jealousy, envy’. However, when her practice faltered, she described it as a “downfall,” a term that evoking moral failure, like a fallen angel moving away from purity. For Viv, emotions such as envy and anger were negative qualities to purge, rather than natural parts of the human experience.

Cleo presented a similar approach of pushing away difficult emotions: ‘I would describe myself as a well. As in very deep, and it takes a lot to release anything’, a metaphor alluding to a deep pain needing to travel a long way to be released. Meditation often dredged up painful memories, including incidents from her childhood that she felt the need to confront: ‘I know, hand on heart… even in my meditation, it's telling me I need to have those conversations with my mother’. She hesitated to address this need out of fear of invalidation ‘(pause) my issue is I don't want her to say because of her issues – that didn't happen’. When painful memories surface, Cleo tries to push them away, explaining that her ‘childhood trauma is in its box, and meditation is about moving forward’. Yet, in a contradictive manner she also acknowledged that ‘you have to unwrap it… we can just put it in a box and you can forget about it and then it will rear its ugly head’. Her unresolved trauma frequently resurfaces, ‘gone through all the grief and I've processed it, but it does come up a lot’, especially during triggers like the experience of the COVID‐19 pandemic, when ‘I'm now triggered to being a 7‐year old child again’. Growing up with a volatile mother, burying emotions in a ‘box’ may have been necessary to survive. Although she uses meditation to ‘move forward’, situations can bring her back to feeling 7 years old again – helpless and in danger.

#### For some, emotions were processed through the body; others kept the body distant, seeing it as temporary or secondary

Participants' approaches to processing emotions often reflected their relationship with their bodies. For Theo, the body has a central role and is ‘connected to emotions’ while meditation is described as: ‘a space for my body to communicate its own intelligence and its own experience. Everything which is being absorbed physically, suppressed or taken on then has an opportunity to make itself heard’. Processing emotions is a thoroughly bodily experience, his ‘whole upper body is kind of contorting a bit’. These sensations of ‘real intense pain’ led to ‘bottoming out and feeling incredible joy’. Meditation helped him reconnect with his body after periods of dissociation, which he likened to having ‘split off some of yourself into a room and closed the door’. Meditation helped him ‘re‐enter’ his body – now ‘your chest fills out and you feel like you're feeling your whole body’. This suggests that Theo had to first release his pain by ‘bottoming out’ through meditation before he was able to unlock a door where part of him lived and feel unified in one ‘whole’ body. Cleo also experienced emotions physically during meditation. She described feeling ‘numb’ and ‘partially paralysed… because you're just so grounded’ a feeling that makes her feel ‘connected… like that pushing it's all gravity’ and feels ‘great’. This feeling of pushing down to the ground could be seen as rooting for the Earth like a tree, a simultaneous deep connection and total surrender that may be healing after growing up with a mother who ‘is not a carer’.

However, for Bill and Viv the body was merely a vessel rather than an essential part of who they are. Bill described his body as: ‘I'm not my body […] my body's just like a vehicle’, a means to an end: ‘I want something that's practical, that goes where I need it to go’. He is aware of his body during meditation in terms of being successful at not feeling pain ‘I can sit cross legged in there for half hour, an hour and it's not a problem’. Interestingly, he also noticed ‘holding on a bit of a lump in my throat, so I can feel that I'm sort of holding maybe some emotion in my throat’. His difficulty in accepting and experiencing more negative emotions seems to manifest physically as a lump: an object of tensity and hardness obstructing breathing, and therefore existing, in ease. Viv similarly devalued the body, influenced by her spiritual beliefs: ‘I don't think that my body can give me sort of what meditation gives me because it's not permanent’. This belief had harmful ramifications on her physical health: ‘I lost like 10‐15 kilograms… every single person I met was telling me like, you're so skinny, like you look unhealthy’, which she waved away as ‘it didn't affect me because I was just like… I'm not this body’.

### Meditation offered a space to explore relating to the self and others, areas complicated by their inconsistent EDN

Participants described their meditation journey as one of deep self‐discovery, reflecting on their relationship with themselves through self‐love and self‐compassion and their relationship with others through moral evaluation and interdependence.

#### Many struggled with self‐love and self‐compassion

Participants reported a journey of self‐exploration due to meditation with the processes of self‐love and self‐compassion being more prominent. Loving themselves was presented as a struggle stemming from their childhood.

Theo realised a need to connect with his inner child: ‘I first understood that I needed to parent myself… some of the grief was coming from a small boy who was very upset and I as the adult recognise that’. This felt gentler than connecting with his adult self: ‘it's possible to feel sympathy for a little boy, but it's not always possible to feel sympathy for your adult self’. He practices self‐compassion by saying to his younger self: ‘you're safe, you don't have to be frightened’. Similarly, Tony described meditation as a way to see past struggles from ‘all angles’, and reconnect with his younger self by going ‘to the years back and… you could see how sad your younger self was’.

Dolores also experienced self‐love through meditation, which helped her break free from her parents' expectations to be ‘the best’. She realised that ‘I don't have to be perfect because no one is’, and began loving herself for who she is, separate from imposed standards: ‘Meditation teaches you how to love yourself in a way because you dive deep into yourself’. Dolores expressed pride in her achievements, indicating a high self‐esteem, but showed some judgement and intellectualisation towards her younger self, saying, ‘I would rather if this younger version wouldn't be so like such a people‐pleaser… But then again, this child didn't know any better, that was a coping mechanism I created, and it worked for the time being’. While admitting that ‘it worked’ there is no compassion shared for the girl who had to people‐please to feel safe.

Dolores described that she hasn't realised she was ‘the victim of domestic violence’ until she filled in our questionnaire, similarly to Bill who initially denied that he was abused, despite his answers in our questionnaire, and then added that ‘at the time, that's kind of normal’, indicating a struggle with self‐compassion. Throughout his interview, whenever Bill mentioned difficult situations that could evoke compassion in the interviewer, he laughed: ‘I didn't have this ideal childhood, and my parents were hardly ever there (laughs)’. When talking as if he were his father he said: ‘I can hurt you. I can become very unpleasant and quite nasty (laughs)’. Laughing off something emotionally difficult may be a defence towards vulnerability, but also a denial of self‐compassion. Near the end of his interview, Bill voiced an ongoing struggle with self‐love, describing it as: ‘still burning in my mind. […] I neglected my own happiness’. Bill mentioned this, unprompted, indicating this topic's acute significance for him, intensified by using the word ‘burning’. He drew on Buddhism teachings to justify his pursuit: ‘I've actually realised that it's OK to be happy […] Buddha taught Tantric teachings as well. So, it's like you can enjoy things and use that as spiritual practice’. This reliance on Buddha for guidance suggests a need for validation, reflecting the lack of emotional support he experienced growing up. His father's anger, which Bill rationalised as a lesson on ‘the dangers of anger’, left him without a positive emotional role model.

Viv shared similar struggles with self‐love due to her childhood, as while she is ‘under some sort of a journey of self‐love’, it didn't come easy as ‘I've always been the person… to give in every single relationship and then I don't give anything to myself’. Like Bill and Dolores, Viv did not express compassion for her difficult experiences in childhood. Instead, she rationalised her parents' behaviour as ‘they had to work’ and felt guilty for the anger she felt in the past as her mum ‘was trying her hardest’.

The participants' difficulties with self‐love and self‐compassion seem linked to their ability to acknowledge and accept the pain they experience in childhood. The guilt may also reflect a resistance against prioritising personal authenticity over the care for attachment figures.

#### While participants found that meditation increased empathy, difficulties with appropriate relational closeness were presented

Meditation led participants to explore morality and closeness to others. Cleo said: ‘in my mind being less full, I then have time to appreciate what others are doing’ and Theo expressed a new depth of affection for friends: ‘I didn't feel how I feel for them now. I felt like affection and I would enjoy their company. But now when I think of them I can feel something glow in my body. Which I would have been kind of cut off from before’. Tony reflected on how his connections shifted from ‘surface level interaction’ to engaging in ‘things that make you feel more connected to people’.

Participants also emphasised a need to establish boundaries post‐meditation. Having grown up with emotionally restrictive parents, Dolores initially struggled: ‘How do you know what you're protecting and where you need to kind of set the boundaries?’, but now ‘I've learned how to voice my feelings and how to be more open about them and not like hide them because I'm scared I will hurt someone else's feelings, because mine are as important as theirs’. Similarly, Cleo described how meditation reinforced her boundaries: ‘the simple act of me meditating every day… instils there is a boundary there and that is my time on my cushion in my space’. She further explained how boundary‐setting strengthened her marriage: ‘I reprioritised myself… And suddenly for him, I became more attractive… we went back to the people that we met 15 years ago’. These insights suggest self‐focus through meditation may bring people closer to their needs as well as foster social awareness and relational sensitivity, benefitting practitioners and close others. The challenge of learning boundaries in adulthood is evident in Theo's description: ‘you're not getting your emotional needs met. For your sense of self, that breeds a real injustice and anger, which you won't be conscious of at that age because you are dependent upon them (meaning parents)’.

The need for protection through, perhaps rigid, boundaries may explain Cleo's tendency to distance herself from others and be self‐reliant. She does not share her pain with others and instead takes on a caretaker role: ‘whilst everybody else is suffering and telling you that they're suffering and […] you know I'm suffering, but you weren't allowed to say that’ because she is seen ‘as the person that they can go to when something happens’. Cleo also describes herself through the metaphor of ‘an island’ and repeats ‘I don't *need* (emphasis by her) anybody’, multiple times throughout her interview. This attitude manifests in her meditation practice which she does solitarily because ‘I felt I was being observed’ by teachers. Yet, she admitted that this tendency to isolate ‘is annoying’, hinting at a desire for connection. Viv similarly resists being ‘dependent on my friends and family, or my relationships’ but seeks fulfilment through a deep connection with Krishna as her ‘source of love and everything I wanted’. Bill expressed a related need, finding spiritual connection in his teacher, saying he ‘always tried to find positive role models’ and would ‘do anything’ his teacher asked.

## DISCUSSION

This study aimed to explore the personal and relational experiences of individuals with a history of EDN‐inconsistent care and meditative practice. The intention was drawn from the premise that the EDN outlines a path of species‐typical care neglected in our recent history, potentially leading to developmental trauma impacting social and relational intelligences, which may become evident in contemplative practices. Indeed, participants described their experience of an inconsistent EDN and subsequent emotional and relational struggles persisting into their present lives. These struggles involved recognising and accepting emotions, regaining embodiment, knowing healthy boundaries, feeling self‐love or self‐compassion, and relating with others in a way that felt safe and fulfilling.

While this study confirmed previous findings related to an inconsistent EDN, such as experiencing anxiety, depression and social withdrawal, this is the first study offering experiential findings on the meaning and effects of an inconsistent EDN. Participants consistently described childhood environments that were emotionally unresponsive, volatile, or both. Common themes included parental absence and/or overwhelming, suffocating caregivers who instilled fear. This dynamic often led to parent–child role confusion, where participants ignored their emotions to tend to their parents' emotional needs. Many withdrew emotionally or physically to protect themselves. They felt their ‘true self’ was unwelcome, instead adopting a ‘perfect child’ persona to avoid rejection or abuse. As supported by current literature, this non‐containing upbringing contributed to participants using neurotic and immature defence mechanisms, such as dissociation, intellectualisation, repression and denial (Lemma, 2015).

Meditation was presented as a respite from inner struggles, anxiety and rumination, by helping participants calm down, in accordance with current literature. Calm was reached by managing repetitive thoughts, first noticing them and then letting them go. However, participants' intention and approach to meditation varied, with some moving towards and others away from accepting all emotions. Three participants consciously welcomed all emotions and tried to enhance their awareness of them, which led to experiencing challenging emotions during meditation, such as anger or grief. These emotions were not described as MRAEs but as part of a releasing process that ultimately brought deep joy.

Other participants, though, aimed to dispose challenging emotions and presented less capacity to recognise or reflect on their emotions, especially vivid in those practising religious meditation. Even though meditation teachings encourage firstly accepting all emotions and subsequently detaching from them, misinterpretation is easy due to moral teachings attached to some religions using meditation practices. For example, in Theravada Buddhism scriptures anger is described as one of ‘mind's impurities’, so ‘one should win anger through kindness’ (Rahula, 2007, pp. 151, 132). Similarly, Bhakti Yoga is described as a ‘cleaning process of negative emotions such as anger, anxiety, contempt, disgust, embarrassment, fear, guilt, offence, sadness etc’. (Maurya et al., 2018, p. 486). Misinterpretations can perpetuate parental invalidation experienced in childhood, reinforcing the notion that ‘bad’ emotions must be suppressed. This notion aligns poorly with modern psychological approaches and the EDN, which view all emotions as valid and emphasise that healthy emotional development relies on parenting that addresses both positive and negative emotions through empathy, provision of emotional relief and reassurance (Narvaez et al., 2016; Thompson, 1994). For people whose emotional worlds were invalidated in childhood, these teachings could exacerbate difficulties, reinforcing patterns of self‐denial and emotional suppression. Therefore, an inconsistent EDN may have obstructed participants from feeling and accepting the full breadth of their emotions, a state that meditation intensified for some participants as they attributed their suppression to spiritual progress.

For most participants there was a connection between the way they processed their emotions and the way they related to their body. Overall, there was a pre‐existing difficulty to connect with the body, as evident in dissociative tendencies. Even though no studies have explored the connection of such symptoms and EDN, children growing up in communities characterised by EDN‐consistent care seem more likely to experience a sense of self that maintains its embodied nature relative to EDN‐inconsistent environments (Ross et al., 2017). Moreover, emotional maltreatment, which could reflect an inconsistent EDN, is associated with dissociation (Ó Laoide et al., 2018). Typically, our bodies do not function in independence; our mind, body and emotions are interconnected, and the way humans perceive, process and regulate emotions is embodied (Damasio & Carvalho, 2013; Herbert & Pollatos, 2012). In agreement with studies, meditation helped participants reduce bodily symptoms of dissociation, but only if they had a welcoming approach towards challenging emotions and ‘allowed’ their bodies to speak (Michal et al., 2007; Zerubavel & Messman‐Moore, 2015). However, the opposite approach of a wilful disconnection from the body, was observed in participants who would not welcome challenging emotions. Therefore, the effects of meditation depended on participants' motivations: those who had a welcoming approach towards their body and emotions experienced an increased connection to both, while a denial of them led to further disconnect.

Participants expressed that meditation led them on a journey of self‐love. Some participants succeeded more in traversing it than others, and some participants specifically highlighted that it is something they struggle to experience. Current literature focuses on meditation's effect on self‐compassion rather than self‐love. Self‐love focuses on finding joy through self‐contact, self‐acceptance and self‐care, while self‐compassion tends to focus on suffering, seen as part of a shared humanity (Henschke & Sedlmeier, 2021). The participant's struggle to experience self‐love may be understandable through the lens of an inconsistent EDN and its impact on self‐development. For a baby to be able to understand and process its emotions, containment by a ‘good enough mother’, meaning a mirroring and active holding, is needed (Waddell, 2018). However, when the carer's mirroring is incongruent and does not represent the infant's internal state, the development of a sense of self is hindered and instead a ‘false self’ is built up, one of low self‐esteem and self‐agency (Narvaez, 2014; Winnicott, 2018). These difficulties could hinder one's capabilities to find joy through self‐contact, self‐acceptance and self‐care, yet, this is an unexplored topic and further studies would be needed in this area.

Participants expressed a belief that their struggle to love themselves was rooted in their childhood. Some participants described an intuitive need to repair this by attending to their ‘inner child’. The concept, initially coined by Jung (1963), has received recognition in popular culture but remains underexplored in academic research. In our study, participants described reparenting themselves by forming a relationship with their inner child, offering emotional and cognitive containment and support that their caregivers were not in a position to provide. Participants reported healing effects from this practice, suggesting its potential as a therapeutic approach to addressing developmental trauma.

Participants also shared going through a socio‐moral exploration, expressing an ongoing ambiguity regarding relating to other people. On the one hand, all participants felt more connected to others, experienced a desire to help others and an increased ability to understand their perspective, consistent with studies showing that meditation leads to increased theory of mind and social connectedness (Hutcherson et al., 2008; Tan et al., 2014). However, participants also described a need to maintain a distance from others, or seeking intense, close relationships with spiritual beings and teachers. People with insecure attachment, which is associated with an inconsistent EDN, may seek emotional security either through counter‐dependence by maintaining distance from other people, or overdependence of inappropriately close relationships (Quick et al., 1992). Social opposition and withdrawal have indeed been found to be associated with an inconsistent EDN (Narvaez et al., 2016) while intense relationships may compensate for the absence of an early caregiving that fulfilled their emotional needs (Dontas et al., 1985; Freud & Dann, 1951). Children that do not grow up with parental presence, synchrony, empathy, mentalising, and play, create a view of a world that is not safe, and therefore self‐protection through social withdrawal and distancing is vital (Narvaez, 2014; Samuels & Pryce, 2008). This need was evident in the complex narratives by participants' need to establish boundaries with other people. Therefore, even though participants stated that meditation helped them feel more connected, as they started their lives in relationships that were deeply unsafe or unfulfilling, a need for either safe distance or closeness that they never had, persisted in their lives and permeated their meditation practice. This may speak to the challenge of experiencing healthy intimacy once this has been violated and distorted.

## CONCLUSION

This is the first study to present personal experiences of adults with an inconsistent EDN. It explored the meditation experiences of six participants with an inconsistent EDN using the IPA method, identifying three key themes: ‘An inconsistent EDN in childhood left lasting emotional and relational wounds into adulthood’, ‘Meditation eased inner turmoil but revealed different ways of dealing with emotions and the body’, and ‘Meditation offered a space to explore relating to the self and others, areas complicated by their inconsistent EDN’. Meditation helped participants find moments of inner calm and foster closer connections with others, but persisting difficulties tied to their childhood experiences remained significant. Participants struggled with accepting emotions, connecting with their bodies, practising self‐compassion, and managing relational boundaries. These challenges were especially pronounced among those engaging in religious meditation.

These findings advance the field by illuminating how early developmental trauma may surface and needs to be re‐negotiated through contemplative practices. Given that participants used meditation as a mental health tool, caution in its teaching and practice is warranted, as it may reinforce rather than alleviate pre‐existing maladaptive psychological processes for some individuals. These findings are particularly valuable for psychologists and psychotherapists, highlighting the importance of trauma‐informed practice, especially in the context of mindfulness‐based interventions.

As with all IPA studies, the aim is not to generalise findings to the broader population, but to offer in‐depth insight into how individuals make sense of their unique experiences. Some limitations are noted, as the study provides a snapshot of participants' experiences, highlighting the need for future research to adopt a longitudinal design to clarify how healing unfolds over time and what practices best support sustainable emotional and relational development. Additionally, the role of external factors – such as personal therapy, protective relationships, and religion – emerged as influential. Intimate relationships, in particular, appeared pivotal in participants' healing, with some narratives underscoring the transformative impact of love and acceptance from partners. This aligns with evidence that relational capacities, although shaped early in life, can evolve through supportive adult relationships. Future research could investigate how relational and contemplative practices might jointly foster healing in individuals with inconsistent EDNs. Insights into these interconnected journeys could enhance the effectiveness of meditation‐based interventions. This underscores the complexity of healing for individuals navigating the aftermath of childhood relational trauma and points to the importance of tailored, integrative approaches.

## AUTHOR CONTRIBUTIONS

**Anna‐Maria Frastali:** Conceptualization; writing – original draft; writing – review and editing; methodology; project administration. **Adhip Rawal:** Supervision; conceptualization; writing – review and editing.

## CONFLICT OF INTEREST STATEMENT

The authors report there are no competing interests to declare.

## Data Availability

The data that support the findings of this study are available from the corresponding author, AMF, upon reasonable request.
